# Dosimetric properties of a newly developed thermoluminescent sheet‐type dosimeter for clinical proton beams

**DOI:** 10.1002/acm2.13222

**Published:** 2021-03-15

**Authors:** Takahiro Kato, Tatsuhiko Sagara, Shinya Komori, Ryohei Kato, Akihiko Takeuchi, Yuki Narita

**Affiliations:** ^1^ Preparing Section for New Faculty of Medical Science Fukushima Medical University Fukushima Japan; ^2^ Department of Radiation Physics and Technology Southern Tohoku Proton Therapy Center Fukushima Japan

**Keywords:** *in vivo* skin dosimetry, proton therapy, quality assurance, thermoluminescent dosimeter

## Abstract

**Purpose:**

This study aimed to evaluate the dosimetric properties of a newly developed thermoluminescent sheet‐type dosimeter (TLD‐sheet) for clinical proton beams.

**Materials and Methods:**

The TLD‐sheet is composed mainly of manganese doped lithium triborate, with a physical size and thickness of 150 mm × 150 mm and 0.15 mm respectively. It is flexible and can be cut freely for usage. The TLD‐sheet has an effective atomic number of 7.3 and tissue‐equivalent properties. We tested the reproducibility, fading effect, dose linearity, homogeneity, energy dependence, and water equivalent thickness (WET) of the TLD‐sheet for clinical proton beams. We conducted tests with both unmodulated and modulated proton beams at energies of 150 and 210 MeV.

**Results:**

The measurement reproducibility was within 4%, which included the inhomogeneity of the TLD‐sheet. The fading rates were approximately 20% and 30% after 2 and 7 days respectively. The TLD‐sheet showed notable energy dependence in the Bragg peak and distal end of the spread‐out Bragg peak regions. However, the dose–response characteristics of the TLD‐sheet remained linear up to a physical dose of 10 Gy in this study. This linearity was highly superior to those of commonly used radiochromic film. The thin WET of the TLD‐sheet had little effect on the range.

**Conclusion:**

Although notable energy dependences were observed in Bragg peak region, the response characteristics examined in this study, such as reproducibility, fading effects, dose linearity, dose homogeneity and WET, showed that the TLD‐sheet can be a useful and effective dosimetry tool. With its flexible and reusable characteristics, it may also be an excellent *in vivo* skin dosimetry tool for proton therapy.

## 1 INTRODUCTION

Proton therapy (PT) is growing at a considerable pace, which is visible in increasing in the number of the installation of new facilities worldwide,^1^ and the related technology continues to evolve. Its irradiation techniques are also diversifying, and there is much debate about quality assurance (QA) methods for managing them. In order to overcome the difficulty in PT QA, the QA tools dedicated to PT has been developed. Similar to photon therapy, the use of two‐dimensional (2D) detectors with excellent real‐time properties is widespread in the field of PT, but the traditional film method is still used. Among the films, radiochromic films that can be handled in a typical room lighting are widely used because of their advantageous properties of tissue‐equivalence, high spatial resolution, self‐developing, and stability after irradiation.^2^ However, there are some precautions for use such as non‐linear dose response characteristics, non‐flexibility, and non‐reusability.^2^, ^3^ EBT3 film has flexibility to some extent and can be curved. But it is hard to hold the shape without maintaining a force applied on it and fit the film on irregular surface.

It has been reported that films are used not only for QA such as 2D dose distribution but also for *in vivo* dosimetry.^4^, ^5^, ^6^ For some treatments, *in vivo* dosimetry is required because treatment planning alone is insufficient to ensure accurate coverage of the target. And conceivably, passive scattering PT (PSPT) often involves a treatment plan with a small number of fields and a high entrance skin dose.^7^, ^8^ By comparing the accurately measured delivered skin dose with the skin reactions, physicians can better quantify the risks of toxic effects in individual patients. Conventional rod‐type TLDs and glass dosimeters can be used, but there is a limit to the evaluation based on points alone from the viewpoint of positioning accuracy. In addition, there is a concern with using conventional dosimeters *in vivo* due to their thickness and effect on the proton range. In recent years, the development of a film with excellent flexibility has been studied as a method for overcoming this problem.^3^

Recently, with the advent of the charge‐coupled device (CCD) camera, the high‐resolution imaging of large areas has become much faster, so the amount of research on photon imaging using 2D thermoluminescent dosimeter (TLD) has been increased.^9^, ^10^, ^11^, ^12^, ^13^, ^14^, ^15^, ^16^, ^17^ Annalakshmi et al. reported that manganese doped lithium triborate has a single grow peak and linear dose response to beta‐ray irradiation up to 50 Gy.^13^ Actually, the basic response characteristics of various thermoluminescent materials to the proton beam have also been studied,^10^, ^11^, ^12^, ^15^ but the reality is that none of them have reached the level of perfection that can be generalized. Therefore, further studies are required. In this study, the dosimetric characteristics of a newly developed TLD‐sheet of manganese doped lithium triborate, LiB_3_O_5_:Mn, were investigated using modulated and unmodulated proton beams.

## 2 MATERIALS AND METHODS

### 2.A Specifications of the thermoluminescent sheet‐type dosimeter (TLD‐sheet)

**Fig. 1:**
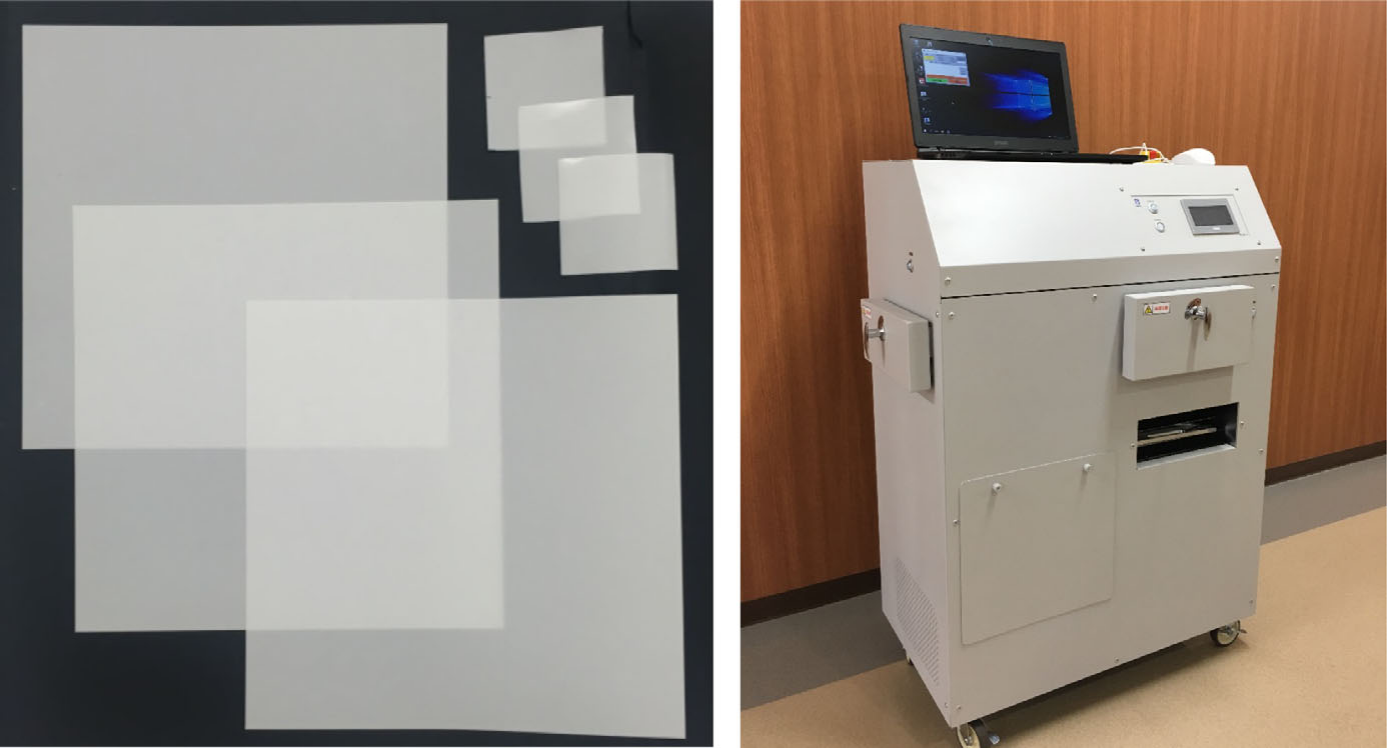
The appearances of various size of the TLD‐sheets (left) and the TLD reader (TLDR‐1) (right). The TLD‐sheet is white and semi‐transparent. Data can be acquired using the TLDR‐1 within 15 min.

The TLD‐sheet and TLDR‐1 analysis reader used in this study was developed by TOYO MEDIC CO. LTD. (Tokyo, Japan). Figure 1 depicts the appearance of them. The TLD‐sheet was composed mainly of manganese doped lithium triborate, LiB_3_O_5_:Mn. The crystal of this phosphor material was grinded and mixed with silicone to form a sheet with a size of 150 mm × 150 mm and can be easily cut into small pieces, if necessary. The physical thickness of the TLD‐sheet was 0.15 mm. The effective atomic number was 7.3, which is a tissue‐equivalent property.^18^ The spatial resolution of the reader was 288 dpi.^19^

### 2.B TLD‐sheet analysis procedures

**Fig. 2:**
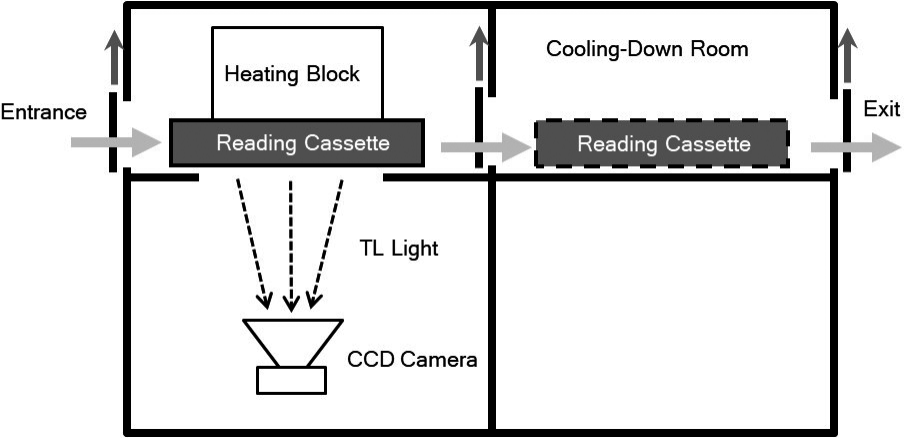
Schematic diagram of the TLD reader. The reading cassette is shown in the measuring position (left) or cooling‐down position (right). After the cassette is inserted via the left entrance, the heating block adheres to the flexible cover of the cassette and increases the temperature of the TLD‐sheet. The thermoluminescence light is detected using a charge‐coupled device camera during the time determined by the user. After the measurement, the reading cassette is transferred to the cooling‐down room until the temperature becomes low enough for safe manual removal.

A schematic diagram of the TLDR‐1 is shown in Fig. 2. The TLDR‐1 was equipped with a CCD camera, heating block with thermometer, and reading cassette carrier. After placing the TLD‐sheet on the glass plate of the reading cassette, which comprised a glass base plate supported by a metal frame and overlaid by a flexible cover, the cassette was inserted into the reader and placed in front of the CCD camera.^19^ The intensity map obtained by CCD camera was corrected using flat fielding method with the dark frame image taken prior to the measurement.^19^ This process eliminates the back ground (signals obtained even when no light exists) and the effect of the pixel‐to‐pixel sensitivity variations. The accuracy of the reader’s response to uniform illumination was evaluated as 0.03% (standard deviation) in the region of maximum TLD‐sheet’s size, and no off‐axis effect of the detector in both up‐down and left‐right directions was observed^19^ in contrast with EBT3 film.^20^ The heating block was maintained at a specified temperature within an accuracy of ±1°C and was used to heat the TLD‐sheet via conduction through the flexible cover of the cassette. The adhesion strength between the heating block and the cassette was finely adjusted to avoid damaging the glass and ensure a consistent TLD‐sheet temperature during the measurements. Thermoluminescent light that penetrated the glass of the cassette was detected using the CCD camera. The measurement time and temperature were adjusted according to the properties of the TLD response. The reader has a pre‐annealing option, wherein a lower temperature is used to remove the signals from lower energy peaks immediately before the measurement to avoid any undesirable fading characteristics. After the measurement, the cassette was moved to a cooling‐down position. This enables consecutive measurements of different TLD‐sheets. The measured intensity images were analyzed using ImageJ software (NIH, Bethesda, MD, USA).

In our measurements, the TLD‐sheet was heated at 200°C for 2 min prior to irradiation to clear any trapped electrons and holes and degas the TLD‐sheet. This process helped us to eliminate non‐irradiation‐related signals, reduce contamination, and obtain a clearer image. The measurement duration was 15 min, and the temperature was maintained at 210°C. No pre‐annealing option was applied.

### 2.C Evaluation of the dosimetric properties of the TLD‐sheet

At our institution, the proton‐type particle therapy system (Hitachi, Kashiwa, Japan) was used for this study. This machine uses the wobbler method, a type of PSPT.^21^ The particle therapy system is comprised of an ion source, a 3‐MeV radiofrequency quadrupole linear accelerator, a 235‐MeV synchrotron, a high‐energy beam transport line, two gantry irradiation rooms, and one irradiation room for the horizontal beam. The proton beam delivery system includes beam‐wobbling magnets, a lead scatterer, a main dose monitor, a ridge filter, a range shifter, a backup monitor, a flatness monitor, a block collimator, a multi‐leaf collimator, a range compensator, and a patient‐specific collimator, if necessary.

Currently, both the TLD‐sheet and TLDR‐1 are under development, and the specific details have not been fully clarified, particularly for proton beams. The following basic response characteristics of the clinical proton beam were examined: reproducibility, fading effect, dose linearity, homogeneity, and energy dependence. Furthermore, we measured the water equivalent thickness (WET) because it is important not to influence the dose distribution when conducting *in vivo* skin dosimetry (IVSD). Under test conditions, we applied unmodulated and modulated proton beams at energies of 150 and 210 MeV. The details of each respective method are described below. All measurements were obtained three times, and the mean values were calculated.

#### 2.C.1 Reproducibility

To verify the reproducibility of the repeated measurements, irradiation was repeated 10 times under the same conditions on different days. The reading value of the sheet was obtained five days after each irradiation. During this period, the reproducibility of the output was evaluated daily using an ionization chamber dosimeter according to our standard protocol, and the results were confirmed to be within ±0.2%. A small piece (30 mm × 30 mm) of the same sheet was placed at the center of the spread‐out Bragg peak (SOBP) on the central axis of a beam with an energy of 150 MeV and SOBP size of 60 mm beam. The irradiation field size was 20 cm × 20 cm. A Solid Water phantom (SUN NUCLEAR, FL, USA) was used. An isocenter was set at the center of the SOBP, and irradiation was administered at a physical dose of 1 Gy. The readings were performed 5 days after irradiation, and the reproducibility of the reading values from the TLD‐sheet axis was evaluated.

#### 2.C.2 Fading effect

The fading characteristics of the TLD‐sheet were evaluated using a proton beam energy of 150 MeV and SOBP size of 60 mm. Four pieces of 30 mm × 30 mm TLD‐sheet were placed at the center of the SOBP of the Solid Water phantom and irradiated with a physical dose of 1 Gy. After irradiation, the TLD‐sheets were stored in a shading bag at a temperature of 23°C to eliminate or reduce optical and thermal fading. The four TLD‐sheets were readout at different times after the completion of irradiation, from 10 min to 7 days. The average intensities were calculated from three measurements, and the intensities were reported relative to the 10 min readout.

#### 2.C.3 Dose linearity

A 30 mm × 30 mm TLD‐sheet was placed in the Solid Water phantom, and irradiation was administered at a physical dose of 0–10 Gy (14 dose points) and a field size of 100 mm × 100 mm. The TLD‐sheet was placed in the plateau region of a 210‐MeV unmodulated beam (depth: 20 mm), which corresponded to a region dominated by high‐energy components, and at the center of the SOBP of a 150‐MeV beam with a 20 mm‐SOBP, which corresponded to a region that included low‐energy components. It has been confirmed by measurement with a dosimeter that the dose distribution uniformity in the area of 30 mm × 30 mm, which corresponds to the size of the sheet, is <1%.

#### 2.C.4 Homogeneity

The comprehensive 2D response characteristics of the TLD‐sheet and TLDR‐1 were examined to access the homogeneity of the reading system. The TLD‐sheet was irradiated perpendicularly to the beam axis in the Solid Water phantom to obtain a two‐dimensional dose distribution in the plateau region and in the center of the SOBP of a 150‐MeV beam with a SOBP size of 60 mm. The field size was 10 cm × 10 cm. The two orthogonal profiles perpendicular to the beam axis were compared with those obtained using a PinPoint3D Ion Chamber (Type 31022; PTW, Freiburg, Germany) and a horizontal beam‐type motorized water phantom (TOYO MEDIC CO. LTD., Tokyo, Japan).

#### 2.C.5 Energy dependence

For a simple evaluation of the energy characteristics, the TLD‐sheet was placed parallel to the beam axis in Solid Water phantoms,^22^ and the percentage depth dose (PDD) was determined. The direction of the beam was horizontal, and the TLD‐sheet was placed between the vertically stacked phantoms (the largest surfaces of the slab phantoms were facing upward/downward). This reduces air gaps between the phantoms by gravitational force compared to the alternative configuration of the phantoms (i.e., the phantoms were standing). Therefore, only the former configuration was used in this measurement. One common practice used to eliminate the air gap effect involves tilting the film plane a few degrees away from the central axis of the beam.^23^, ^24^ We tilted the film plane 3° away from this central axis when using both a 150‐MeV unmodulated beam and a 150‐MeV beam with a SOBP size of 60 mm. The maximum TLD‐sheet dimensions were 150 mm × 150 mm. Range shifter was used so that the range of proton beam becomes shorter than the TLD‐sheet size, and this enabled us to measure entire dose profile using a single TLD‐sheet. The PDD results were measured under the same conditions using an Advanced Markus chamber (Type 34045; PTW, Freiburg, Germany) with a horizontal beam‐type motorized water phantom. These PDDs were then compared.

#### 2.C.6 WET

The PDD was measured with and without a stack of the TLD‐sheet of 30 mm × 30 mm attached to the front side of the beam injection surface of a horizontal beam‐type motorized water phantom. The water equivalent thickness was derived from the difference in the PDD range according to the presence or absence of a stack of sheets. To improve the measurement accuracy, a stack of 10 sheets was used, and the water equivalent thickness per sheet was determined. Usually, a TLD‐sheet is combined with a removable protective polyethylene terephthalate sheet. However, the TLD‐sheet with the protective sheet is not flexible enough. The protective sheet can be removed easily as opposed to radiochromic film, which has a structure of phosphorous layer tightly sandwiched between hard protective layers.^2^ Therefore, we measured the WET only without the protective sheet. For reference purposes, the experiment under exactly the same conditions as TLD‐sheet was conducted using EBT3 radiochromic film (Ashland, NJ, USA) as a control, and the results were compared.

## 3 RESULTS

**Fig. 3:**
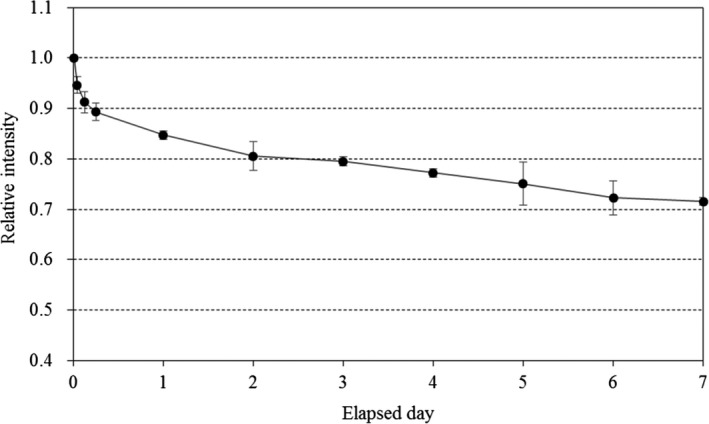
Fading characteristics of the TLD‐sheet. The proton energy was 150 MeV, and the sheets were placed at the center of the spread‐out Bragg peak. The error bar indicates the standard deviation.

**Fig. 4:**
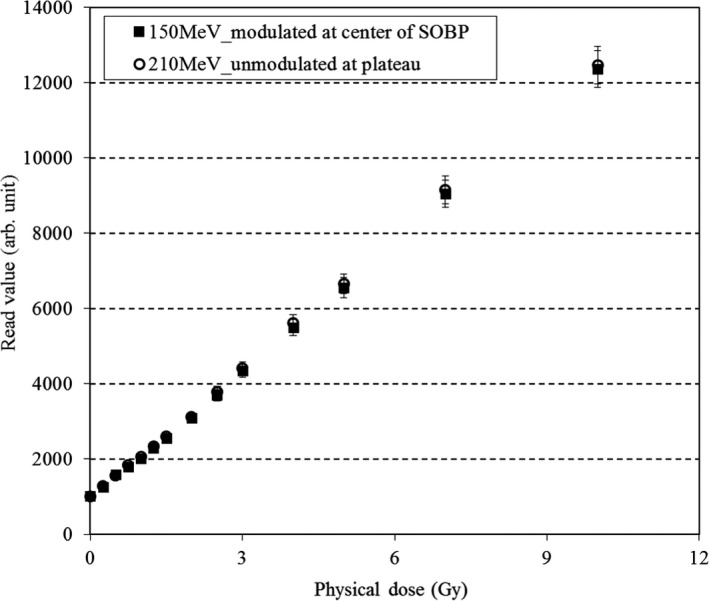
Dose linearity of the TLD‐sheet. The two different symbols represent measurements obtained in the plateau region of a 210‐MeV unmodulated beam and at the center of the 150‐MeV beam with a spread‐out Bragg peak (SOBP) size of 20 mm. The error bar indicates the standard deviation.

**Fig. 5:**
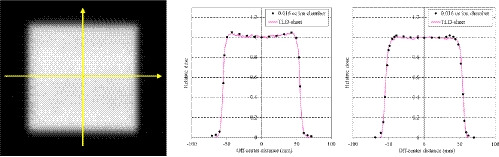
Sample 2‐dimensional image of the TLD‐sheet at the plateau region of the 150‐MeV beam with a spread‐out Bragg peak size of 60 mm (left). Normalized horizontal profile (middle) and vertical profile (right) through the center of the TLD‐sheet. The directions are shown as yellow arrows on the left image and comparison with those measured with the PinPoint 3D Ion Chamber is shown.

**Fig. 6:**
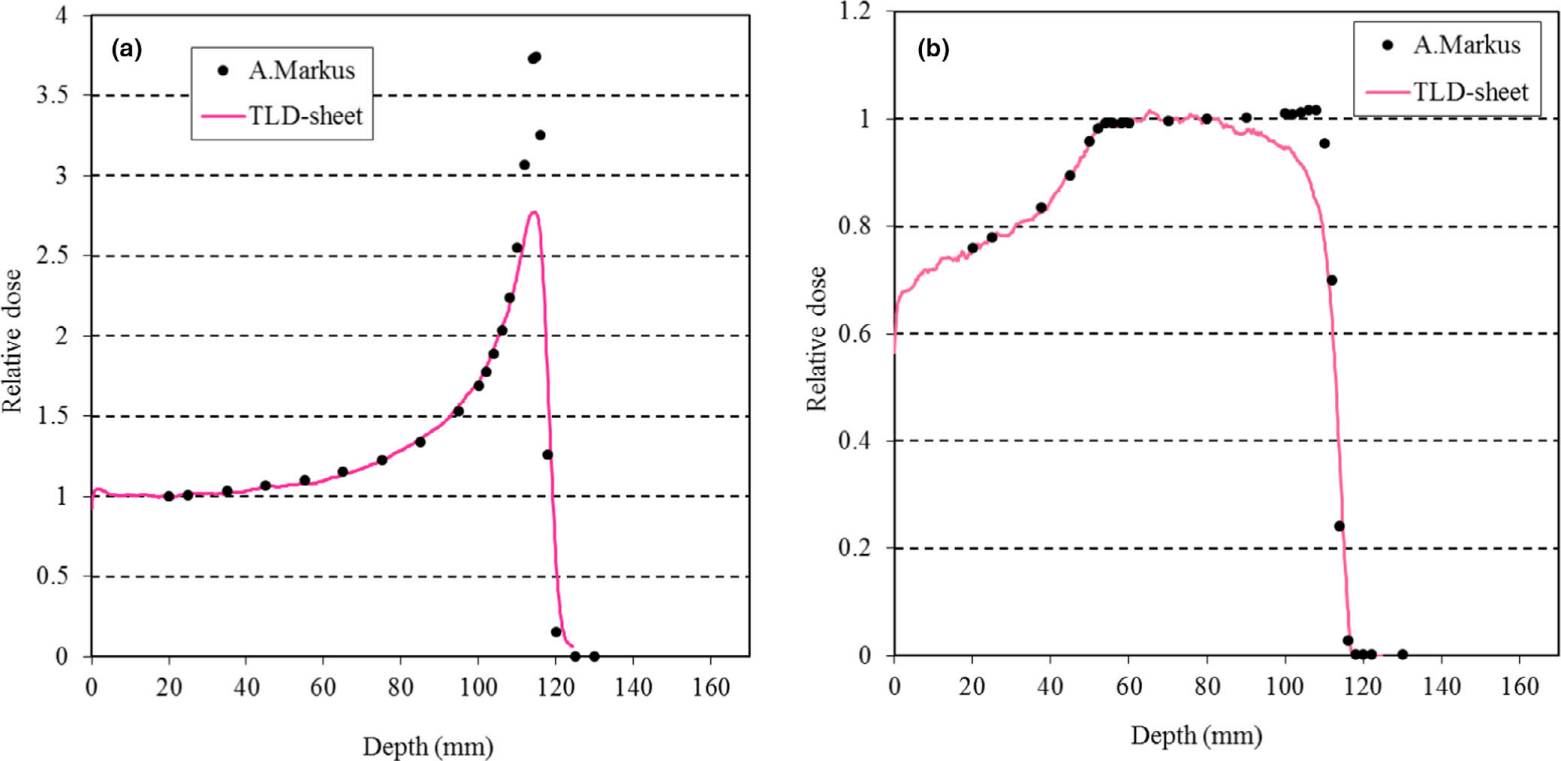
Comparison of (a) the unmodulated and (b) modulated percentage depth doses obtained using the TLD‐sheet and the Advanced Markus (A. Markus) chamber.

The results of the 10 repeated measurements obtained under the same irradiation conditions on different days indicated that the system yielded a readout reproducibility within 4% (coefficient of variation = 2.7%). Figure 3 presents the fading curve of the TLD‐sheet. A normal fading decay was observed, with fading rates of approximately 20% and 30% after 2 and 7 days respectively. Figure 4 presents the dose–response characteristics. We confirmed that linearity was maintained up to a physical dose of at least 10 Gy. The measured values in the SOBP center of a 150‐MeV beam with a SOBP size of 20 mm tended to be slightly lower than those in the plateau region (i.e. 20 mm depth) of a 210‐MeV unmodulated beam, likely due to the energy‐dependence of the TLD material, but the differences were within 2%. Figure 5 presents the dose profiles obtained at the plateau region of the 150‐MeV beam with a SOBP size of 60 mm as a reference. A median filter was used to reduce noise, as described for the EBT3 analysis.^25^ Thereafter, we confirmed the measurement at each depth was highly consistent with the measurement obtained in the PinPoint3D Ion Chamber. The maximum differences between the two measurements were within 2%. Figure 6 presents the results of the PDD measurements. The PDDs for the unmodulated and modulated beams were normalized at the plateau region and the center of the SOBP respectively. We confirmed that the TLD‐sheet exhibited a non‐linear response in the Bragg peak region. We also observed poor response characteristics on the TLD‐sheet at the distal‐end of the SOBP region. Finally, the WET of the TLD‐sheet and EBT3 were 0.22 and 0.36 mm respectively. The corresponding nominal physical thickness values were 0.15 and 0.27 mm respectively. For both, the WETs were consistent with <0.1 mm from the physical thickness.

## 4 DISCUSSION

We evaluated the dosimetric properties of a newly developed TLD‐sheet using a newly developed TLDR‐1 reader for clinical proton beams. Although we observed a slight energy dependence of 2% between 150 and 210 MeV and notable poor response in the Bragg peak region and distal end of the spread‐out Bragg peak regions, the response characteristics such as the reproducibility, fading effect, dose linearity, homogeneity, and water equivalent thickness showed that this newly designed TLD‐sheet can be a useful dosimetric tool for PT. Our examination of reproducibility as a basic response characteristic confirmed an accuracy level within 4%. The TL intensity was sharply faded 6 h after irradiation and gradual decay occurred after approximately 2 days after irradiation. Maruyama et al. reported that TL intensity with their Cr‐doped Al_2_O_3_ thermoluminescent slab dosimeter was sharply faded 24 h after irradiation and gradual decay occurred after 100 h.^16^ Therefore, the TLDS we used in this study is considered to have slightly improved fading characteristics compared to their results. In actual clinical situations, an evaluation within 2 days may be necessary. However, it is important to set an appropriate wait‐time window for the evaluation, as it is done after EBT3 measurement.^26^ The dose linearity of the TLD‐sheet was significantly superior to that of widely used EBT3 and recently released EBT‐XD (Ashland, NJ, USA).^27^ The dose–response for the TLD‐sheet was verified up to 10 Gy, within the clinical practice dose limits of PT in Japan.^28^ Based on the reports of Annalakshmi et al., it may be possible to sufficiently evaluate the linearity up to even higher dose ranges.^13^ This approach may be effective for the verification of single‐dose irradiation methods, such as stereotactic radiosurgery with volumetric modulated arc therapy,^29^ and we will continue to verify this possibility in the future studies.

Regarding homogeneity, across the 10 cm × 10 cm profile, the TLD‐sheet measurement points were consistently within 2% of those obtained using the PinPoint3D Ion Chamber, suggesting that the TLD‐sheet could also be applied in the context of machine quality assurance (QA). Compared to film, the TLD‐sheet has the advantage of reusability. However, many aspects of durability, such as sheet degeneration caused by repetitive heat application, remain unclear, and further testing is needed. Furthermore, TLD‐sheets do not exhibit visible changes, unlike radiochromic film, and therefore the results cannot be verified immediately after irradiation. In this regard, radiochromic film has the advantage of providing an immediate qualitative image.

Previous analyses of the dose–response characteristics in film and TLD to the proton beam energy suggested that the responses to low‐energy proton beams might be reduced.^10^, ^11^, ^15^, ^23^, ^24^ This phenomenon has been widely attributed to a quenching effect that occurs with an increased linear energy transfer (LET) along an incident particle track.^30^, ^31^ Additionally, a higher initial energy level would result in a higher mean energy level at the Bragg peak, as well as a larger energy spread, which would result in a lower average LET. Hence, the effect can change depending on the beam configuration. In this study, the verification was limited to simple conditions, but in future studies, we plan to examine the LET dependency in detail. The effects of oblique incidence are also considered to be an issue for future study.

Regarding the WET, the nominal physical thickness and actual measured WET were consistent to within 0.1 mm. We confirmed that TLD‐sheet and EBT3 have tissue‐equivalent properties by checking the equivalence of physical thickness and WET and thus very little impact on the range. This is considered to be the thinnest 2D TLD reported so far.^10^, ^11^, ^12^, ^14^ Although the EBT3 has thicker WET, the difference is approximately 0.1 mm, and EBT3 can also be used in medical dosimetry.

EBT3 involves a combination of protective material,^32^ which reduces its flexibility in the shape. Removal of the protective covering on the TLD‐sheet allows for more flexibility compared to EBT3. This approach is expected to be particularly effective for IVSD in the head and neck region, where the contour shape is complicated. The energy and angular dependencies in superficial region are important factors for the use in IVSD and have to be clarified. We will investigate these topics in detail in the future work. In addition, it is known that damage can occur typically in 1 to 2 mm from the edge of EBT film, when it is cut by scissors.^33^ Even though this damage can be minimized by following the instruction provided by the vender,^34^ bending it will damage the edge. This damage is significant for small size films. By contrast, the TLD‐sheet does not have a layered structure, and the phosphor material is uniformly distributed in silicone. No damage can be caused by cutting the TLD‐sheet. Therefore, new TLD‐sheet has an advantage over the EBT film in this respect. The comparative advantages of the TLD‐sheet are the very thin size, reusable nature, and easy resizing by free cutting. Although the use of the TLD‐sheet generally requires some time due to the indirect readout, it could be used practically without any issues if the optimal conditions that enable an accurate analysis can be determined.

Some aspects of the TLD‐sheet remain under development. Therefore, we think that further studies of various aspects, such as the optimum temperature setting during measurement, are warranted. Furthermore, during IVSD, the TLD‐sheet may be cut into small pieces. Not only superior dose response characteristics but also the ability to cut the TLD‐sheet into any size is the greatest advantage of this technology. Therefore, we considered that a further examination of the light emission detection accuracy when the sheet is cut into small pieces is also necessary.

## 5 CONCLUSION

We evaluated the dosimetric properties of a newly developed TLD‐sheet for clinical proton beams. Although notable energy dependences were observed in the Bragg peak region, the response characteristics such as the reproducibility, fading effect, dose linearity, homogeneity, and water equivalent thickness showed that this newly designed TLD‐sheet can be a useful dosimetric tool for PT. Moreover, we confirmed that the dose linearity of the TLD‐sheet was highly superior to those of the EBT3 and EBT‐XD. In addition to these results, TLD‐sheet can be considered promising due to its excellent flexibility and reusability. These preliminary results suggest that the TLD‐sheet is a useful and effective dosimetric tool not only for machine QA but also IVSD in PT.

## CONFLICT OF INTEREST

The author declares that we have no conflict of interest.

## AUTHOR CONTRIBUTION

Takahiro Kato: Conceptualization, Investigation, Writing–Original Draft. Tatsuhiko Sagara: Resources, Methodology. Shinya Komori: Formal analysis. Ryohei Kato: Formal analysis. Akihiko Takeuchi: Validation. Yuki Narita: Writing–Review & Editing.
